# Psp140: an immunodominant antigen in the supernatant of *Streptococcus pneumoniae* culture

**DOI:** 10.18502/ijm.v12i4.3938

**Published:** 2020-08

**Authors:** Davoud Afshar, Solmaz Ohadian Moghadam, Farhad Safarpoor Dehkordi, Reza Ranjbar, Amir Hasanzadeh

**Affiliations:** 1 Department of Microbiology, School of Medicine, Zanjan University of Medical Sciences, Zanjan, Iran; 2 Uro Oncology Research Center, Tehran University of Medical Sciences, Tehran, Iran; 3 Halal Research Center of IRI, FDA, Tehran, Iran; 4 Molecular Biology Research Center, Systems Biology and Poisonings Institute, Baqiyatallah University of Medical Sciences, Tehran, Iran; 5 Department of Microbiology, Maragheh University of Medical Sciences, Maragheh, Iran

**Keywords:** Enzyme-linked immunosorbent assay, Immunodominant antigen, *Streptococcus pneumoniae*, Western blot

## Abstract

**Background and Objectives:**

*Streptococcus pneumoniae* causes many lethal infections. Due to its reduced sensitivity to commonly used antibiotics, development of new strategies against pneumococcal infections seems to be necessary. We aimed to investigate immunodominant antigens in *S. pneumoniae* culture supernatant in order to develop novel targets for pneumococcal vaccines.

**Materials and Methods:**

In this study *S. pneumoniae* ATCC49619 was sub-cultured into BHI broth from overnight culture at 37°C for 4 h. The supernatant proteins were precipitated using acetone precipitation method. A rabbit was intramuscularly immunized with alum adjuvant and 100 μg pneumococcal supernatant proteins, 6 times at 14 days’ intervals to produce hyperimmune serum. ELISA assay was performed to determine the antibody level response to pneumococcal secretory proteins. Then dot blot applied for rapid evaluation of hyperimmune serum reactivity to pneumococcus supernatant proteins. The western blot was also used to determine the interaction of supernatant proteins with immunogenic rabbit’s hyperimmune-serum.

**Results:**

According to the western blot analysis, the immunodominant protein had 140KDa molecular weight and designated as pneumococcal secretory protein140 (Psp140).

**Conclusion:**

The Psp140 protein in the supernatant of *S. pneumoniae* culture is an immunodominant protein and it is likely related to pneumococcal secretory protein or surface exposed protein which released into culture supernatant during bacterial growth.

## INTRODUCTION

*Streptococcus pneumoniae* is a Gram-positive bacterium that causes many life-threating infections including meningitis, sinusitis, otitis media and septicemia (1–4). In addition to these diseases, the bacterium can lead community-acquired pneumonia, which is the leading cause of death especially in children under 5 years of age and kills 1.6 million people annually (5, 6). It is reported that in the United States about 5% of patients with pneumococcal pneumonia die from the disease (7). Due to the reduced sensitivity of *S. pneumoniae* to commonly used antibiotics, development of new strategies against pneumococcal infections appears to be essential (8).

There are currently two pneumococcal vaccines available including a 23-valent polysaccharide vaccine (PPV23) and three conjugate vaccines: 7-valent (PCV7), 10-valent (PCV10) and 13-valent (PCV13). These vaccines are recommended for high-risk groups including patients with chronic diseases, chronic obstructive pulmonary disease (COPD), cochlear implants, cranial-injured patients, smokers, diabetes and immunosuppressive patients (9). The PPSV23 and PCV13 are used in children and adults of 5 to 64 years old. However, polysaccharide vaccines do not elicit protective immune responses in children < 2 years old. As a result, conjugate vaccines have been introduced in order to resolve this limitation (10).

The immunogenic secreted pneumococcal proteins interact with the host immune system and stimulate the immune cells to produce antibody. It has been well recognized that several well-known virulence factors are released in pneumococcal secretome including PspC, PrtA, PsaA, PbpA, PhtD, AmiA, ZmpB, Eno and Ply proteins (11).

In the present study, we aimed to investigate immunodominant antigens in the pneumococcal culture supernatant so that they can be used as immunostimulants in order to developing novel targets for vaccines against pneumococcal infections.

## MATERIALS AND METHODS

### Bacterial culture supernatant analysis.

*S. pneumoniae* ATCC49619 was cultured into brain heart infusion (BHI) broth (HiMedia, India) and incubated at 37°C for 24 h. Then, 2 mL from overnight culture was sub-cultured into 50 BHI broth and incubated at 37°C for 4 h. The medium was subsequently centrifuged at 9000 rpm for 3 minutes. The supernatant proteins were then precipitated using acetone precipitation method and the results were electrophoresed on 10% polyacrylamide gel. The concentration of supernatant proteins was also measured by spectrophotometry method using Nano drop instrument with following formula: (Protein concentration (mg/ml) = 1.55A280 – 0.75A260).

### Induction of immunity in rabbit.

To prepare hyperimmune serum against pneumococcal secretome, a male rabbit with weight of 2–3kg was purchased from Pasteur Institute of Iran. The rabbit was intra-muscularly immunized with alum adjuvant and 100 μg supernatant proteins for six times at 14 days’ intervals (12, 13). For evaluation of humoral immunity responses against pneumococcal supernatant proteins, non-immune and last hyperimmune rabbit sera were checked by enzyme-linked immunosorbent assay (ELISA). Briefly, the concentrated supernatant proteins were dissolved in Phosphate-buffered saline (PBS), 100 μl (10 μg/mL) of it was transferred into 96-well polystyrene plates and incubated overnight at 4°C. The wells were washed 3 times with washing buffer (PBS containing 0.05 Tween20) and blocked with PBS containing 4% skimmed milk for 1 hour at room temperature. The wells were washed 3 times with washing buffer, hyperimmune and non-immune rabbit sera were separately added into the wells at dilution of 1:100 to 1:12800, and incubated for 1 h at 25°C. After that, 100 μl anti-rabbit IgG conjugated with peroxidase was added into the wells and incubated for 30 min at 25°C. Then, 3, 3′, 5, 5′-tetramethylbenzi-dine (TMB) was added into the wells and the reaction was terminated by 50 μl of 1N H2SO4 after 10 min.

### Dot blot assay and western blotting.

Dot blot and western blot assays were applied for the evaluation of hyperimmune serum reactivity to pneumococcus secretory proteins. In the dot blot, 20 μg secretory proteins were added onto Polyvinylidene fluoride (PVDF) membrane and after 1 h, the membrane was blocked in phosphate-buffered saline (PBS) containing 0.05% Tween 20 and 4% skimmed milk at room temperature for 2 h. Then, 200 μl rabbit hyperimmune serum (at 1:50 dilution) was added onto membrane and incubated for 2 h at room temperature and after washing with phosphate-buffered saline (PBS) containing 0.05% Tween 20 (PBST), the membrane subjected to anti-rabbit antibody conjugated to horseradish peroxidase (HRP) (at 1:1000 dilution) for 1 h at room temperature. Finally, the membrane was treated with 3, 3′-diaminobenzidine solution (Sigma-Aldrich, USA) for 3 min.

For western blot assay, the secretory proteins were electrophoresed in 10% polyacrylamide gel and transferred into PVDF membrane. The membrane was blocked in phosphate-buffered saline (PBS) containing 0.05% Tween 20 and 4% skimmed milk at room temperature for 12 h and washed three times with PBST. The membrane incubated with rabbit hyperimmune serum (at 1:50 dilution) for 2 h and after washing with PBST, subjected to anti-rabbit antibody conjugated to horseradish peroxidase (HRP) (at 1:1000 dilution) for 1 h at room temperature. Subsequent to washing three times with PBST, the membrane was treated with 3, 3′-diaminobenzidine solution (Sigma-Aldrich, USA) for about 10 min. Non-immune rabbit serum and a lane without secretory proteins were used as negative controls in both tests.

## RESULTS

**Fig. 1.**
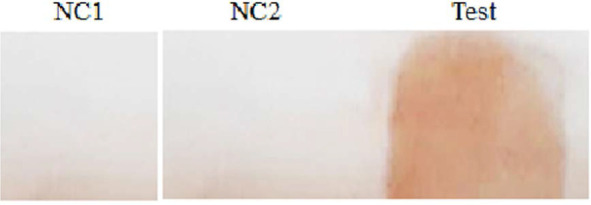
Titration of rabbit non immune serum and hyperimmune serumafter last secretory proteins injection. The numbers 1–8 are 1:100, 1:200, 1:400, 1:800, 1:1600, 1:3200, 1:6400 and 1:12800 sera dilutions, respectively.The vertical line show optical density (OD).

**Fig. 2.**
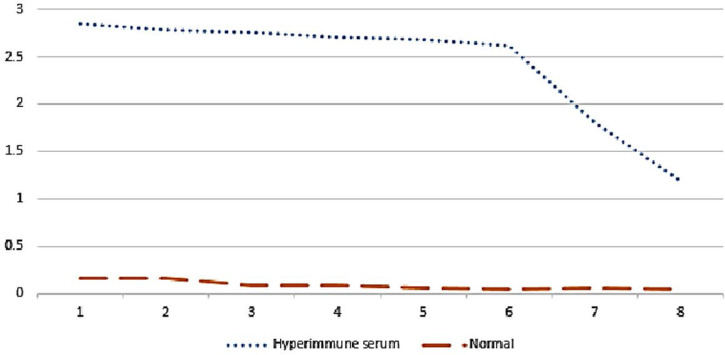
Immunoblotting pneumococcal secretory proteins. NC1; Negative control with non-hyperimmune reaction, NC2; Negative control without secretory proteins and test lane a positive reaction with rabbit hyperimmune serum.

ELISA assay showed that there is an acceptable level of antibody against pneumococcal secretory proteins or proteins following last immunization (Fig. 1). For rapid evaluation of hyperimmune serum reactivity to pneumococcus secretory proteins, dot blot applied and it showed a positive reaction as shown in Fig. 2.

**Fig. 3.**
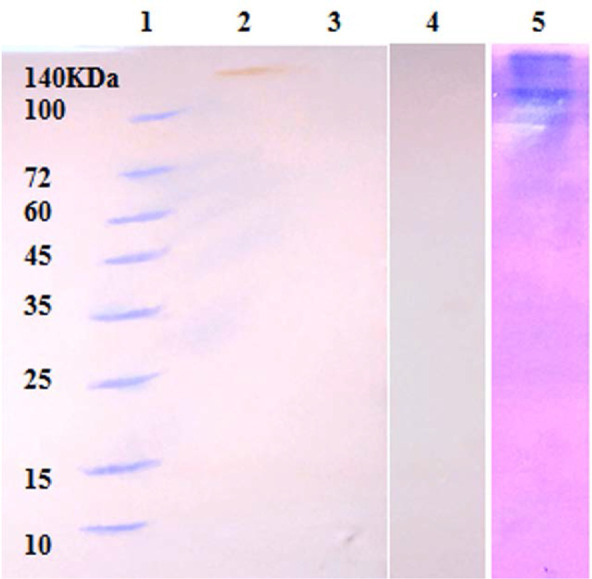
Western blot analysis of secretory proteins of *S. pneumoniae* by hyperimmune serum. lane 1; protein marker, lane 2; the interaction of secretory proteins and rabbit’s hyperimmune serum, lane 3 negative control without secretory proteins, lane 4 negative control with rabbit non-immune serum, lane 5; polyacrylamide gel electrophoresis of secretory proteins.

The western blot was also used to determine secretory proteins and revealed an immunogenic interaction with rabbit’s hyperimmune-serum. The results showed a typical reaction in∼ 140KDa as shown in Fig. 3.

## DISCUSSION

*S. pneumoniae* produces many proteins which play several functional roles for bacterium and in the many cases they interact to immune system protein. Among the bacterial proteome, secretory proteins have a signal peptide at the N-terminal region and secretes by specific systems in the bacterium environment. In the pneumococci, cell autolysis usually happens following bacterium routine incubation and hence, both of secretory and cell wall related proteins can be found in the pneumococcus secretome (14). In the present study, we incubated *S. pneumoniae* culture only for 4 h that caused the cell autolysis did not occur. Therefore, all of the proteins which injected into rabbit belonged to secretory proteins and as shown in the results, only one detectable bond observed in the western blot. One of the possible reasons is the production of one secretory protein at the period of 4 h growth that causes one protein induce immune system and react with hyperimmune serum.

Other hypothesis is that the concentrations of blend proteins were different and the stimulation of immune system has been affected by different proteins concentrations. Although, in a study revealed that the immunogenicity of the secretory proteins was not proportional to the abundance of pneumococcal proteins (11).

Another reason may be resulted from this fact that the immune system, from several secretory proteins, just induced against one protein which is immunodominant antigen. The immunodominant antigens in the *S. pneumoniae* have been previously described (15).

The immunodominant antigens are selected proteins in antigens complex and induce immune system more than other antigens. These antigens mostly have a high molecular weight and in many cases, they have complicated structures. As shown in western blot analysis, the Psp140 has a molecular weight ∼140KDa, which consider as a weighty antigen among pneumococcal proteins.

From several proteins in the pneumococcal secretome and regarding the molecular weight of Psp140, it seems that this protein probably belongs to neuraminidase protein family. Three pneumococcal neuraminidases, NanA, NanB and NanC, which have been previously described, are implicated in pneumococci pathogenesis (16). Janapatla et al. showed that the neuraminidases as major virulence factors are potential targets for pneumococcal vaccine candidates because of immunization with these antigens increases survival of mice challenged with different *S. pneumoniae* strains (17).

When the pneumococci are grown in medium culture, some of their cell wall anchored or surface proteins may separate from bacterium surface and release into medium (18). In a study, Roger et al. described a pneumococcal surface-exposed protein known as Streptococcal pullulanase (SpuA) which had 143KDa molecular weight and its existence also confirmed in the supernatant of *S. pneumoniae* culture fluid (19). Considering this subject, one of probabilities about the source of Psp140 is that it is a surface-exposed protein that released into medium fluids during bacterium growth.

In conclusion, the Psp140 protein in the supernatant of *S. pneumoniae* culture fluid with molecular weight ∼140KDa is an immunodominant protein and it is likely related to pneumococcal secretory or surface exposed proteins.
